# Diet and Physical Activity as Mediators of Weight Loss in a Digital Prostate Cancer Survivorship Intervention for Men: Secondary Analysis of the PC-PEP Randomized Trial

**DOI:** 10.1177/15579883261457532

**Published:** 2026-08-05

**Authors:** Becky Power, Nathan K. Smith, Gabriela Ilie, Ricardo A. Rendon, Ross Mason, Andrea Kokorovic, Cody MacDonald, Nikhilesh Patil, David Bowes, Greg Bailly, Derek Wilke, Elizabeth Langley, Robert Rutledge

**Affiliations:** 1 Department of Urology, 12361 Dalhousie University, Halifax, NS, Canada; 2 Department of Community Health and Epidemiology, 12361 Dalhousie University, Halifax, NS, Canada; 3 Department of Radiation Oncology, 12361 Dalhousie University, Halifax, NS, Canada

**Keywords:** cancer survivorship, prostate cancer, digital health, patient empowerment, weight los

## Abstract

Weight gain during prostate cancer treatment can worsen treatment-related side effects, impair quality of life, and increase risks of recurrence and prostate cancer–specific mortality. The Prostate Cancer Patient Empowerment Program (PC-PEP), a comprehensive, home-based 6-month digital intervention, has demonstrated improvements in diet, physical activity, and weight. This secondary analysis examined whether changes in diet and physical activity mediated weight loss among participants receiving PC-PEP. In a randomized clinical trial, 128 men with localized prostate cancer were assigned to PC-PEP (n=66) or standard care (n=62) for six months. Diet quality was assessed using a 12-item summary score reflecting weekly frequency of key food groups. Physical activity level was self-reported as not very active, moderately active, or very active. Mediation models were adjusted for age, comorbidity, treatment modality, time from randomization to treatment, relationship status, medication for anxiety or depression, and baseline weight, diet, and physical activity. The results show that weight loss in the PC-PEP group was significantly mediated by both diet and physical activity, accounting for 21% and 16% of the total effect, respectively. The direct effect of the intervention remained significant. These results indicate that improvements in diet and physical activity partially mediated weight loss in men receiving PC-PEP, while a substantial direct effect suggests that additional components of the intervention contribute to outcomes. Findings highlight the value of comprehensive, multi-faceted digital survivorship interventions for men with prostate cancer and suggest that observed mediation effects are conservative estimates given the use of brief self-reported behavioral measures.

## Key Messages

1. PC-PEP, a 6-month, home-based, multidomain digital survivorship program, improved diet quality and physical activity among men undergoing curative-intent prostate cancer treatment.
2. Improvements in diet quality and physical activity partially mediated the intervention’s effect on weight loss, accounting for 21% and 16% of the total effect, respectively, and 33% jointly in the parallel mediation model.
3. A substantial direct effect remained, indicating that additional behavioural and psychosocial mechanisms beyond diet and physical activity likely contribute to PC-PEP’s weight-related benefits.

## Introduction

Prostate cancer is the second most common malignancy among men worldwide and represents a major contributor to the global burden of men’s health–related morbidity despite excellent survival rates for localized disease (Raychaudhuri et al., 2025; Sung et al., 2021). Curative treatments such as radical prostatectomy and radiotherapy prolong survival (Hamdy et al., 2023) but frequently result in persistent urinary, sexual, bowel, hormonal, and psychological sequelae that diminish quality of life (Al Hussein Al Awamlh et al., 2024; Donovan Jenny L. et al., 2016; Hu et al., 2024; Ilie et al., 2020).

Weight gain during and after treatment is an additional concern. Androgen deprivation therapy (ADT) leads to adverse changes in body composition (Galvão et al., 2021; Smith et al., 2002), and higher body mass index, central adiposity, and post-diagnosis weight gain are associated with increased risks of biochemical recurrence, disease progression, and prostate cancer–specific and cardiovascular mortality (Bhindi et al., 2014; Joshu et al., 2011; Marshall & Joshu, 2020; Troeschel et al., 2020). These patterns highlight the need for behavioural interventions that support sustainable, long-term weight management during survivorship.

Although exercise and dietary modification improve physical function, body composition, and quality of life in men with prostate cancer (Oczkowski et al., 2021; Wilson et al., 2021), adherence is generally low. Only half of Canadian adults meet physical activity recommendations (Statistics Canada, 2021), and dietary quality remain suboptimal (Hack et al., 2020). Qualitative research identifies limited knowledge, low confidence, insufficient social support, and treatment-related symptoms as common barriers to healthy behaviour change (Bergengren et al., 2020; Siapno et al., 2024; Stone et al., 2019; Yannitsos et al., 2020).

Structured resistance and aerobic training improve strength, fitness, and selected quality-of-life outcomes in men on ADT (Galvão et al., 2010), and structured exercise programs after chemotherapy yield clinically meaningful benefits in other cancer populations (Courneya et al., 2025). Yet evidence indicates that exercise-only interventions are insufficient for sustained change. A recent systematic review and meta-analysis reported that improvements in body composition and quality of life are greatest when exercise is embedded within comprehensive behavioural, nutritional, and psychosocial frameworks (Kempin et al., 2024). These findings underscore the need for multi-component survivorship interventions that address the diverse behavioural and psychosocial challenges experienced by men with prostate cancer.

The Prostate Cancer Patient Empowerment Program (PC-PEP) was developed to meet these multidomain needs (Ilie et al., 2023). This six-month, home-based digital intervention delivers daily video-guided content integrating physical activity, dietary guidance, pelvic floor muscle training, stress-reduction practices, and relationship and social-support modules. Secondary analyses of the randomized trial show improvements in weight, diet quality, and physical activity (MacNevin et al., 2024), with additional evidence demonstrating reductions in psychological distress mediated by self-efficacy and illness perceptions (MacDonald et al., 2024), improvements in urinary function (Lawen et al., 2024), and health-system cost-effectiveness (Nuyens et al., 2025). Collectively, these findings suggest that PC-PEP engages multiple behavioural and psychosocial mechanisms.

Despite these observed behavioural improvements, it remains unclear whether changes in diet or physical activity are the pathways through which PC-PEP influences weight loss. Clarifying these mechanisms is essential, as mediation analyses can illuminate how complex digital survivorship interventions exert their effects and guide refinement and scaling. Mediation studies remain uncommon in prostate cancer survivorship research, and mechanistic evidence is limited across lifestyle and digital health interventions.

This analysis examines whether diet quality or physical activity at six months mediate the association between study group (PC-PEP versus waitlist control) and weight change. Understanding these pathways may deepen our knowledge of how multi-component digital survivorship interventions influence behavioural and weight outcomes in men and inform the design and scaling of effective men’s health interventions during and after cancer treatment.

## Materials and Methods

### Study Design and Participants

This secondary analysis used data from a previously published crossover randomized controlled trial. Between December 2019 and January 2021, men with biopsy-confirmed localized prostate cancer scheduled for curative treatment were recruited through urology and radiation oncology clinics or through self-referral in response to clinic advertisements. Eligibility criteria included age ≥18 years, suitability for low-to-moderate physical activity as confirmed by the study physician, ability to attend assessments at baseline, six months, and twelve months, daily internet and email access, and English literacy.

**Figure 1.**
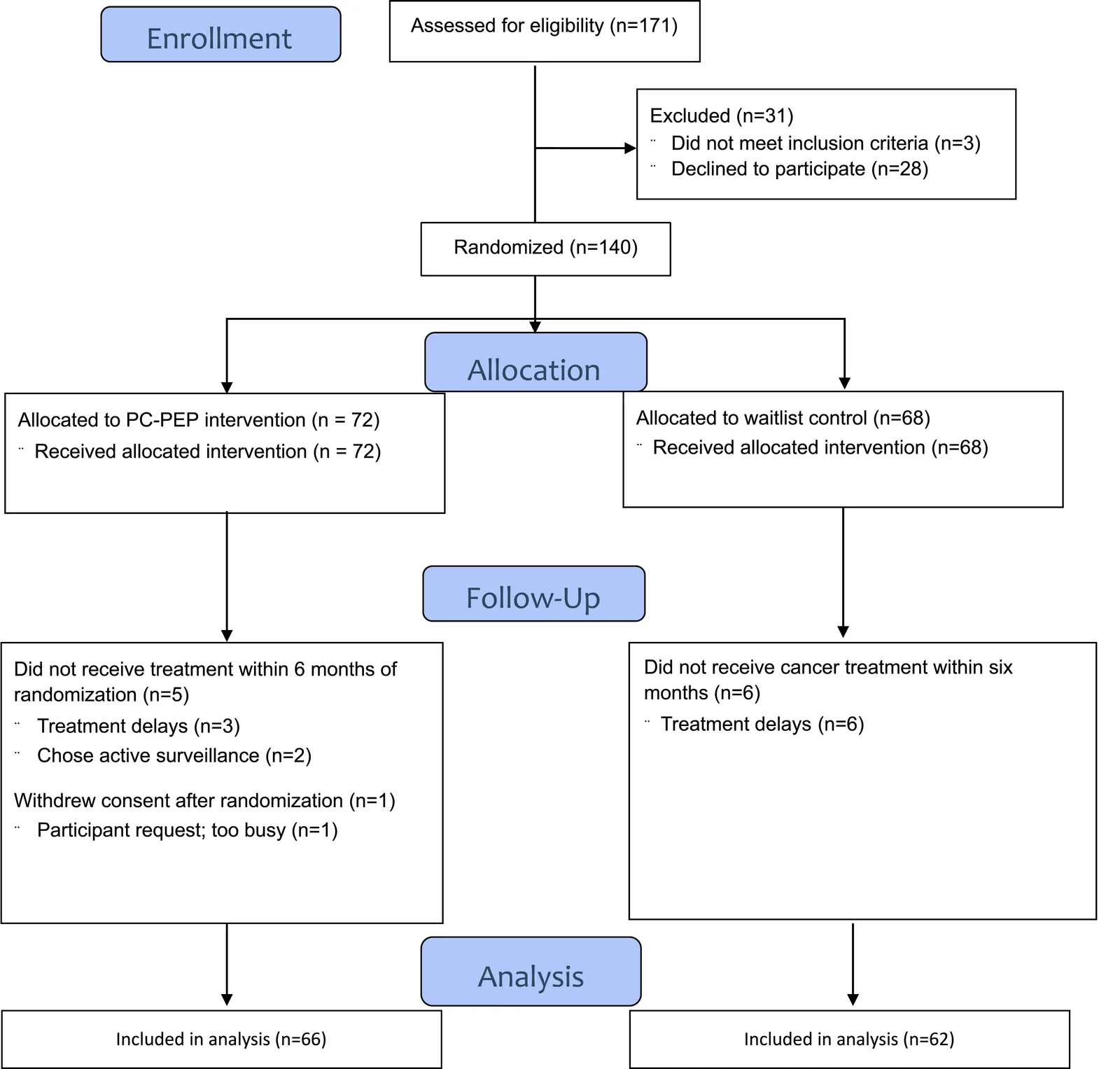
CONSORT 2010 participant flow diagram for the PC-PEP randomized controlled trial. Notes: CONSORT = consolidated standards of reporting trials. PC-PEP = prostate cancer patient empowerment program

Of 171 screened individuals, 140 were randomized 1:1 to PC-PEP or a waitlist control using fixed-block randomization stratified by baseline psychological distress (Figure 1). Research staff collecting data or managing participants were blinded to allocation. One participant withdrew before initiating the intervention, and eleven did not undergo planned curative treatment within six months, yielding a final analytic sample of 128 participants. At six months, the waitlist control group crossed over to receive PC-PEP, and all participants completed follow-up assessments at twelve months.

All participants provided written informed consent, and the study received institutional research ethics approval. Full protocol details and CONSORT-aligned procedures have been published previously.

### Group Exposure: PC-PEP vs Waitlist Control

Participants assigned to PC-PEP received daily emails for six months, each containing a three-to five-minute video providing motivational support and outlining recommended activities. The intervention included stress-reduction practices, nutritional guidance, structured aerobic and strength training, pelvic floor muscle training, and content addressing relationships and intimacy. Participants were encouraged to follow healthy eating practices aligned with Canada’s Food Guide, emphasizing plant-based foods and limiting red meat consumption (Health Canada, 2019). Weekly compliance surveys promoted engagement. The waitlist control group received standard care during the initial six-month period. Detailed descriptions of the exercise and dietary components are available elsewhere (MacNevin et al., 2024).

### Outcome: Weight

Weight (kilograms) was self-reported through online questionnaires at baseline, six months, and twelve months. Self-reported values were used for analysis as planned in-person assessments were disrupted by COVID-19 restrictions. Agreement between available in-person and self-reported weights was excellent (r = 0.99).

### Mediator Variables

#### Physical Activity Level

At baseline, six months, and twelve months, participants classified their physical activity as not very active (<30 minutes of aerobic activity per week), moderately active (≥30–150 minutes/week of moderate activity), or very active (>150 minutes/week of moderate activity, ≥75 minutes/week of vigorous activity, or an equivalent combination).

#### Diet Summary Scores

Diet was assessed at baseline, six months, and twelve months using a twelve-item questionnaire capturing weekly frequency of consumption of key food groups (e.g., vegetables, fruits, fast foods, sweets). Items were scored on 0–3 or 0–5 scales, with higher total scores indicating poorer dietary quality. The measure aligns conceptually with the *Starting the Conversation* brief dietary assessment tool (Paxton et al., 2011), which assesses similar dietary behaviours, and incorporates additional items consistent with Canada’s Food Guide (Health Canada, 2019), including plant-based proteins and healthier fats. A full description of the items and scoring procedures is provided in Supplemental Table S1.

### Prognostic Covariates

Covariates selected a priori based on prior literature included age, Charlson Comorbidity Index, time from randomization to treatment, treatment modality (radical prostatectomy vs radiotherapy), relationship status, and use of prescribed medication for anxiety or depression (Cartwright et al., 2016; Harju et al., 2018; Kurian et al., 2018; Song et al., 2023; Sun et al., 2012; Wu et al., 2017).

### Statistical Analysis

Analyses were performed using SPSS Statistics version 29.0.2.0 (IBM Corporation, 2021). Descriptive statistics summarized baseline characteristics and weight, diet, and physical activity outcomes. Continuous variables are reported as medians with interquartile ranges and categorical variables as frequencies and percentages. Baseline group differences were assessed using Mann–Whitney U tests for continuous variables and chi-square tests for categorical variables. Statistical significance was defined as a two-sided alpha of 0.05.

Mediation analyses evaluated whether diet or physical activity at six months mediated the association between study group and weight. Analyses used PROCESS macro version 5.0 (Model 4) (Hayes, 2021; Rijnhart et al., 2017; Vo et al., 2020), estimating the effect of study group on the mediator, the effect of the mediator on weight, the direct effect of study group on weight, and the total effect. Indirect effects were calculated as the product of the relevant regression coefficients. Separate mediation models were run for diet and physical activity. Moderated mediation analyses using PROCESS Model 7 examined whether treatment modality modified indirect effects (Hayes, 2021; Rijnhart et al., 2017; Vo et al., 2020). All models adjusted for prespecified prognostic covariates and baseline values of both mediators and outcomes, consistent with methodological recommendations (European Medicines Agency, 2015). Indirect effect significance was assessed using bias-corrected bootstrapped confidence intervals based on 10,000 resamples.

### Sensitivity Analyses

Two sensitivity analyses were performed. First, a parallel mediation model including both diet and physical activity simultaneously assessed their combined contribution to the association between study group and weight. Second, mediation analyses evaluated whether diet or physical activity mediated the association between PC-PEP timing (early vs late delivery) and weight change, following prior findings suggesting slightly greater weight loss when PC-PEP was received before primary treatment (MacNevin et al., 2024).

## Results

Baseline characteristics were well balanced between groups (Table 1). No significant differences were observed in demographics, comorbidity, treatment modality, ADT use, time to treatment, cancer risk category, baseline PSA in the salvage subgroup, or use of medication for anxiety or depression (all p>0.3). Baseline weight, diet score, and physical activity level were also similar (all p>0.6).

**Table 1.** Baseline Demographic, Clinical, and Behavioural Characteristics of Curative-Intent Prostate Cancer Patients Randomized to the PC-PEP Intervention or Waitlist Control Group in the Randomized Controlled Trial (n = 128)

​	PC-PEP^a^ n = 66	Waitlist control n = 62	Test statistic	p
*Demographics*				
Age (years)	66 (60, 70)	68 (61, 72)	U=1780.5	0.2
Education, university or greater	31, 47%	37, 60%	X^2^=2.073	0.15
Employment, (full or part time)	22, 33%	23, 37%	​	0.7
Household Income at baseline, >$30,000 CAD/past year	54, 82%	52, 84%	X^2^=0.095	0.8
Relationship Status (currently in a relationship)	59 (89%)	61 (98%)	Fisher’s Exact Test	0.063
Charlson Comorbidity Index	0 (0, 1)	0 (0, 1)	U=1946.5	0.6
Treatment Modality	​	​	​	0.6
RP^b^	29 (44%)	33 (53%)	​	​
RT^c^	27 (41%)	27 (44%)	​	​
Salvage RT^d^	10, (15%)	2, 3%	​	​
Prescribed ADT^e^	27, 41%	21, 34%	​	0.4
Time Between Randomization and RP or RT (including salvage) Treatment (days)	61 (34, 99)	73 (29, 101)	U=1895.5	0.5
Cancer Risk Category^f^ (RP^b^+primary RT^c^±ADT^e^)	​	​	​	0.6
Low	1, 1.5%	2, 3.2%	​	​
Intermediate	42, 75%	40, 67%	​	​
High	13, 23%	18, 30%	​	​
PSA (ng/ml) (salvage group only)	0.11 (0.065–0.16)	0.28 (0.18–0.37)	​	0.5
Prescribed medication for anxiety, depression or both	12 (18%)	7 (11%)	X^2^=1.201	0.3
*Baseline Mediator and Outcome Scores*				
Weight (kilograms)	85 (78, 96)	84 (77, 95)	U=1933.5	0.6
Diet Sum Score	13 (11, 16)	13 (10, 17)	U=2002.5	0.8
Physical Activity	​	​	X^2^=0.275	0.9
Not Very Active (<30 min. per week)	11 (17%)	14 (23%)	​	​
Moderately Active (30-150 min. per week)	33 (50%)	27 (44%)	​	​
Very Active (150+ min. per week)	22 (33%)	21 (34%)	​	​

*Notes*. Continuous variables are reported as median (interquartile range), and categorical variables as n (%).

^a^Prostate Cancer – Patient Empowerment Program.

^b^Radical prostatectomy.

^c^Radiation therapy.

^d^Salvage Radiation Therapy; the radiation therapy and salvage radiation groups were pooled together in the statistical analyses reported to allow for meaningful comparisons.

^e^Androgen Deprivation Therapy.

^f^National Comprehensive Cancer Network (NCCN).

At 6 months, median weight did not differ between groups (84 [78–92] kg vs 84 [77–95] kg; U=1941, p=0.6), but the change in weight from baseline to 6 months did. Participants in the PC-PEP group lost more weight than those in the waitlist control group (median change: -1.37 kg [IQR: -5.12 to 0.86] vs 0.02 kg [IQR: -1.94 to 1.57]; U=1453.5, p=0.005). It should be noted that the mean weight loss was higher than the median, with the PC-PEP group losing a mean of 2.74 kg from a mean baseline of 88.91 kg, corresponding to an average weight loss of 3.1%. This highlights a skewed distribution where heavier individuals at baseline tended to lose more weight by 6 months (Pearson’s r = 0.292, p = 0.017).

Lifestyle outcomes also favoured the intervention. A greater proportion of PC-PEP participants reported being very active at 6 months (52% vs 34%), with fewer being inactive (9% vs 23%; *X*^2^=6.173, p=0.046). Diet summary scores, where lower values indicate healthier dietary choices, were significantly better in the PC-PEP group (median 11 [IQR: 9–14] vs 14 [IQR: 10–16]; U=1365, p=0.001).

**Figure 2.**
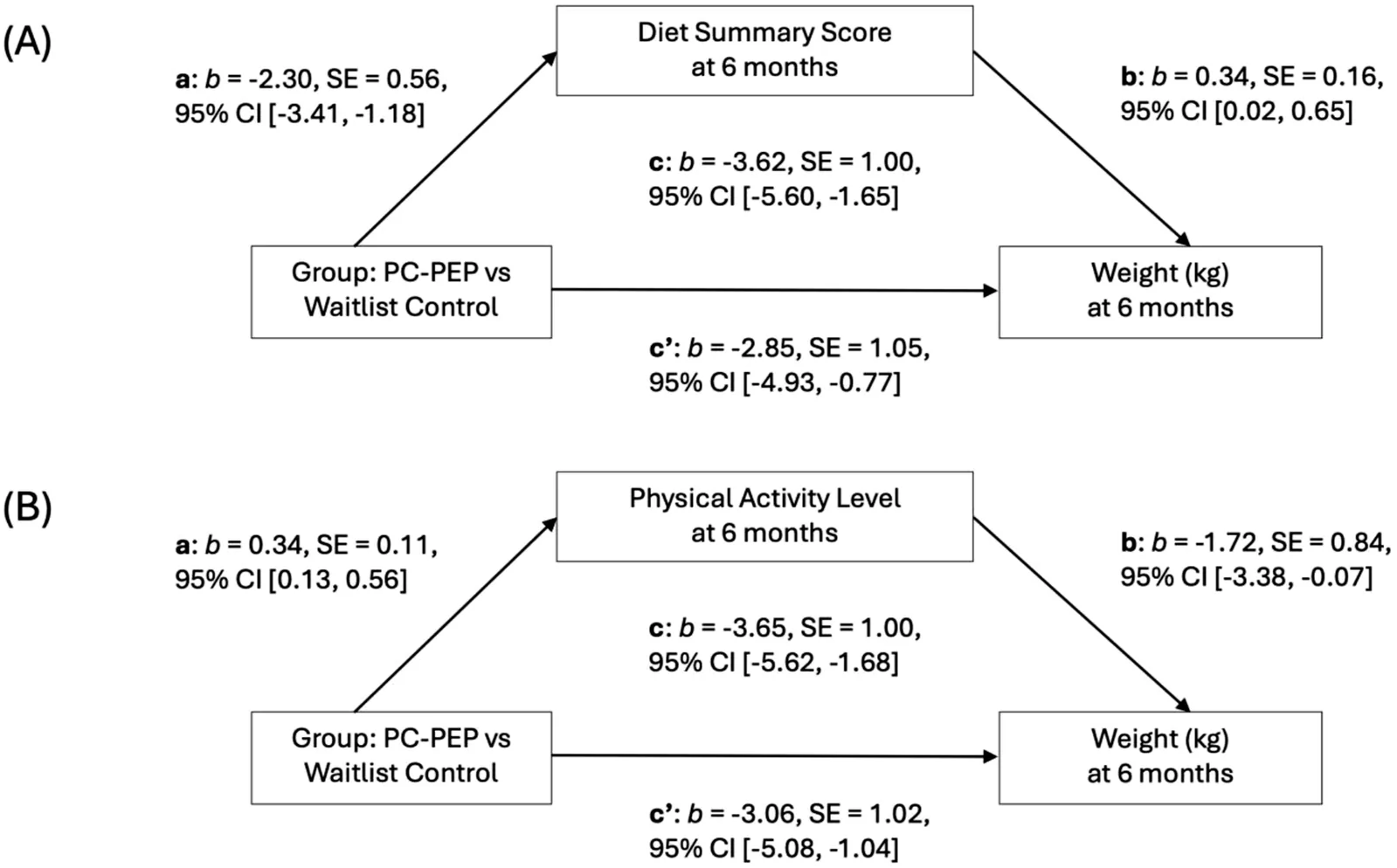
Mediation Pathways Linking PC-PEP to Weight at 6 Months: (A) Diet Summary Score, (B) Physical Activity Level. *Notes.* b = regression coefficient; SE = standard error; CI = confidence interval. Models were adjusted for age, Charlson Comorbidity Index, treatment modality, time between randomization and treatment, relationship status, medication for depression or anxiety, baseline weight, and the corresponding baseline mediator. n = 128 for both models

Mediation analyses were conducted to evaluate whether diet and physical activity at 6 months mediated the association between study group (PC-PEP vs waitlist control) and weight (Table 2; Figure 2). In models adjusted for prognostic covariates and baseline values of weight and the respective mediator, both diet and physical activity significantly mediated the association between study group and weight at 6 months. Diet summary score produced a statistically significant indirect effect of −0.77 kg (95% CI −1.95 to −0.05), accounting for 21% of the total effect. Physical activity level produced an indirect effect of −0.59 kg (95% CI −1.45 to −0.13), accounting for 16% of the total effect. In both models, the direct effect of study group remained significant, indicating partial mediation.

**Table 2.** Mediation Effects of Diet Quality and Physical Activity at 6 Months on the Association Between PC-PEP Participation and Weight Among Curative-Intent Prostate Cancer Patients Enrolled in the Prostate Cancer Patient Empowerment PROGRAM (PC-PEP) Randomized Controlled Trial (n = 128)

Mediator	Total				Direct				Indirect		% Mediated
	Effect size (95% CI)	SE	t	p	Effect size (95% CI)	SE	t	p	Effect size (95% CI)	SE	
Diet Summary Score	-3.62 (-5.60, -1.65)	1.00	-3.63	<0.001	-2.85 (-4.93, -0.77)	1.05	-2.71	0.008	-0.77 (-1.95, -0.05)	0.47	21%
Physical Activity Level	-3.65 (-5.62, 1.68)	1.00	-3.67	<0.001	-3.06 (-5.08, -1.04)	1.02	-2.99	0.004	-0.59 (-1.45, -0.13)	0.32	16%

*Notes.* SE = standard error. CI = confidence interval. Mediation analyses were adjusted for prognostic covariates (age, Charleson Comorbidity Index, treatment modality, time between randomization and treatment, relationship status, medication for depression or anxiety), baseline weight, and baseline diet or physical activity (for the respective analysis). n = 128 for both analyses.

*Moderated mediation analyses* (Table 3) showed no evidence that treatment modality (radical prostatectomy vs radiotherapy, including salvage radiotherapy) modified the mediation pathways for diet or physical activity; all interaction confidence intervals crossed zero.

**Table 3.** Moderated Mediation Effects of Treatment Modality on Diet and Physical Activity Pathways Linking PC-PEP Participation to Weight at 6 Months Among Curative-Intent Prostate Cancer Patients in the Prostate Cancer Patient Empowerment Program (PC-PEP) Randomized Controlled Trial (n = 128)

Mediator	Effect size	SE	Lower 95% CI	Upper 95% CI
Diet Summary Score	0.17	0.45	-0.43	1.45
Physical Activity Level	0.08	0.44	-0.74	1.11

*Notes.* SE = standard error. CI = confidence interval. Moderated mediation analyses were adjusted for prognostic covariates (age, Charleson Comorbidity Index, treatment modality, time between randomization and treatment, relationship status, medication for depression or anxiety), baseline weight, and baseline diet or physical activity (for the respective analysis). n = 128 for both analyses.

A *parallel mediation sensitivity analysis* assessed whether diet and physical activity jointly mediated the association between study group and weight (Supplemental Table S2; Supplemental Figure S1). The combined indirect effect was statistically significant (−1.21 kg; 95% CI −2.49 to −0.30), explaining 33% of the total effect. In contrast, when both mediators were included in the model simultaneously, the individual indirect effects were no longer statistically significant (diet: −0.70 kg, 95% CI −1.84 to 0.03; physical activity: −0.52 kg, 95% CI −1.45 to 0.08). The direct effect remained significant, indicating that diet and physical activity together did not fully account for the observed weight loss (Supplemental Table S2).

A final *sensitivity analysis* examined whether timing of program delivery (early vs late PC-PEP) was mediated by diet or physical activity (Supplemental Table S4). Descriptive pre- and post-intervention values for early and late intervention groups are shown in Supplemental Table S3. There was no evidence of a total effect of intervention timing on weight, nor any significant indirect effects through diet, physical activity, or weekly aerobic or strength training minutes; all confidence intervals crossed zero.

Together, these findings indicate that improvements in diet and physical activity partially mediate the association between study group and weight loss. Additional mechanisms beyond these behavioral pathways likely contribute to the intervention’s effect, and differences in timing of intervention delivery relative to primary treatment did not alter these relationships.

## Discussion

This secondary analysis of the Prostate Cancer Patient Empowerment Program (PC-PEP) randomized trial shows that a comprehensive, home-based digital intervention produces clinically meaningful weight loss in men undergoing curative-intent prostate cancer treatment, and that improvements in diet quality and physical activity partly explain this effect. Diet mediated 21% of the intervention effect on weight and physical activity mediated 16%, with a combined indirect effect of 33% in the parallel model. The direct effect remained statistically significant in all models, indicating that approximately two thirds of the total effect operate through additional mechanisms. Partial mediation is expected in complex behavioral interventions, particularly when mediators are measured with limited granularity and when interventions simultaneously target multiple psychological and behavioral processes. This pattern is consistent with PC-PEP’s multidimensional biopsychosocial design, which integrates daily guidance on nutrition, physical activity, pelvic floor muscle training, stress reduction, and relationship and intimacy support.

On average, participants in the PC-PEP group lost 3.1% of their baseline weight, which meets the commonly used threshold for clinically meaningful weight loss of 3% (Foley et al., 2016; Jensen et al., 2013). Though this weight loss was not uniform across the cohort, those who entered the program with a higher weight tended to lose more, suggesting that the program more strongly benefits those who need it most.

These findings extend prior mechanistic work from the same trial. A previous mediation analysis demonstrated that improvements in self-efficacy alone accounted for over 50% of the program’s effect on psychological distress, with illness perceptions contributing additional mediation (MacDonald et al., 2024). In contrast, improvements in urinary and sexual function, although clinically meaningful, did not mediate changes in distress, yielding indirect effects near zero (Lawen et al., 2024; Salman et al., 2025). These studies indicate that mental health benefits arise primarily from shifts in cognitive and motivational processes rather than from symptom recovery alone. The present results show that diet and physical activity function as active behavioural pathways for weight change but do not fully explain the effect, suggesting that the same empowerment-related, patient activation processes that support psychological outcomes may contribute to sustained behaviour change (Anderson & Funnell, 2005; Dixon et al., 2009).

These mechanisms likely influence how dietary and physical activity behaviors are initiated, sustained, and enacted over time rather than representing pathways independent of behavior itself. The structure of PC-PEP aligns with this mechanistic profile. Participants receive daily video-based instruction that breaks down complex goals into achievable actions, models home-based strength and aerobic training, demonstrates simple dietary strategies, and progressively increases expectations. This format supports key sources of self-efficacy, including mastery experiences, social modelling, and verbal persuasion, consistent with observed increases in self-efficacy in prior analyses (MacDonald et al., 2024). By combining physical activity, nutrition, stress management, and pelvic floor muscle training with content addressing relationships and intimacy, the program simultaneously targets practical and psychological barriers that are well-documented in men with prostate cancer, including limited skills, low confidence, fatigue, and fear of movement (Bergengren et al., 2020; Ilie et al., 2023; Kempin et al., 2024; Siapno et al., 2024; Stone et al., 2019; Yannitsos et al., 2020). Although delivered as a structured six-month program, PC-PEP was designed as a sustained survivorship resource. Monthly video-conference groups continue after the six-month period, and participants may repeat the program as a refresher, supporting long-term maintenance of behavioural change and the cognitive processes that underpin it.

External evidence supports the value of such a multidomain approach. Randomized trials have shown that combined resistance and aerobic training improve strength, fitness, and selected quality-of-life domains in men receiving androgen deprivation therapy (Galvão et al., 2010). Structured exercise following chemotherapy also yields significant benefits in other cancer populations (Courneya et al., 2025). A recent systematic review and meta-analysis showed that the greatest improvements in body composition and quality of life occur when exercise is embedded within broader behavioural, nutritional, and psychosocial frameworks (Kempin et al., 2024). The mediation effects observed in the present analysis indicate that a digitally delivered, comprehensive intervention can produce meaningful changes in diet and physical activity within such a framework. PC-PEP has additionally demonstrated real-world scalability: a recent health economic evaluation found it to be cost-effective from a health system perspective (Nuyens et al., 2025), and the program is currently undergoing implementation testing in Canada, Europe, South Africa, and New Zealand.

The results map directly onto major Canadian guideline recommendations. The Canadian Cardiovascular Harmonized National Guideline Endeavour identifies healthy eating, regular physical activity, maintenance of healthy body weight, stress management, and smoking cessation as foundational therapies for cardiovascular disease prevention and management (Jain et al., 2022). The guideline emphasizes that even modest increases in walking can produce clinically meaningful reductions in body weight, body mass index, and waist circumference, and stresses the need for coordinated lifestyle interventions in patients with clustered risk factors. The behavioural pathways identified in this analysis directly reflect these priorities. Given that prostate cancer affects more than 1.4 million men worldwide each year (Sung et al., 2021), scalable intervention that support behavioural modification have substantial public health relevance.

Similarly, the Canadian Urological Association guideline on androgen deprivation therapy highlights the significant cardiometabolic burden associated with treatment, including increased adiposity, loss of lean mass, dyslipidemia, insulin resistance, and heightened cardiovascular risk, and recommends systematic cardiometabolic assessment and structured resistance and aerobic training to mitigate these adverse effects (Kokorovic et al., 2021). The present findings show that a structured, home-based digital program can deliver the behaviours recommended in these guidelines and generate quantifiable improvements in diet, physical activity, and weight that are relevant for men receiving or anticipating androgen deprivation therapy.

Sensitivity analyses indicated that intervention timing, before versus after primary treatment, did not materially alter behavioural outcomes or mediation effects. Combined with earlier work showing only modest differences in weight loss by timing (MacNevin et al., 2024), these findings suggest that structured digital lifestyle support can be initiated flexibly across the treatment continuum, even in the context of treatment-related fatigue, urinary symptoms, or anxiety that often impede physical activity.

### Clinical Perspective

These results underscore the importance of integrating structured survivorship programs into routine prostate cancer care. They provide quantitative mechanistic evidence that improvements in diet and physical activity are substantive pathways through which the intervention influences weight, a factor linked to prostate cancer progression and cardiovascular mortality (Bhindi et al., 2014; Joshu et al., 2011; Marshall & Joshu, 2020; Troeschel et al., 2020). They also demonstrate that PC-PEP is tightly aligned with national guidelines for cardiovascular and metabolic risk reduction and for management of androgen deprivation therapy–related adverse effects, while offering a practical means of implementation in settings without supervised exercise programs or oncology-specific dietetic support.

### Limitations

Physical activity was assessed with a three-category self-report measure that does not capture intensity or duration, which may underestimate the benefits of structured resistance and aerobic training. The diet summary score, although demonstrating expected associations with weight change, has not been validated against external indices (Health Canada, 2019; Paxton et al., 2011). Weight and behavioural measures were largely self-reported due to COVID-19 pandemic restrictions, although available in-person measurements showed excellent concordance. Both the mediators and outcome were assessed at 6 months, precluding our ability to establish temporal ordering of these factors, and thus limiting causal inference. As a single-centre Canadian trial requiring English literacy, internet access, and attendance at in-person assessments, generalizability may be limited. As with all mediation analyses, the intervention was randomized but mediators were not; residual confounding cannot be excluded. As a result, the proportion of the intervention effect mediated by diet and physical activity is likely underestimated rather than overstated.

## Conclusion

The PC-PEP improved diet quality and physical activity and produced clinically meaningful weight loss, with diet and activity jointly mediating one third of the intervention effect and a substantial direct effect consistent with its biopsychosocial design. These findings support integrating structured, home-based digital survivorship programs into standard prostate cancer care as a scalable men’s health strategy as a practical approach to improving cardiometabolic health, reducing treatment-related burden, and delivering guideline-aligned lifestyle therapy at scale.

## Supplemental Material

Supplemental Material - Diet and Physical Activity as Mediators of Weight Loss in a Digital Prostate Cancer Survivorship Intervention for Men: Secondary Analysis of the PC-PEP Randomized Trial Supplemental Material for Diet and Physical Activity as Mediators of Weight Loss in a Digital Prostate Cancer Survivorship Intervention for Men: Secondary Analysis of the PC-PEP Randomized Trial by Becky Power, Nathan K. Smith, Gabriela Ilie, Ricardo A. Rendon, Ross Mason, Andrea Kokorovic, Cody MacDonald, Nikhilesh Patil, David Bowes, Greg Bailly, Derek Wilke, Elizabeth Langley and Robert Rutledge in American Journal of Men’s Health.

## Data Availability

Data from this study are available to researchers through a data access process in compliance with patient privacy requirements and the Personal Health Information Act, subject to Nova Scotia Health Research Ethics Board approval.*
