# Supplementary material for: Diet and Physical Activity as Mediators of Weight Loss in a Digital Prostate Cancer Survivorship Intervention for Men: Secondary Analysis of the PC-PEP Randomized Trial
Source: Am J Mens Health. 2026 Aug 5;20(4):15579883261457532. doi: 10.1177/15579883261457532 (PMC13504590; doi:10.1177/15579883261457532)
Supplement: Supplementary file 1 [file sj-pdf-1-jmh-10.1177_15579883261457532.pdf]

Supplementary Materials

Diet Summary Score: Development and Conceptual Basis

The twelve-item dietary questionnaire used to generate the diet summary score was developed for the Prostate Cancer Patient Empowerment Program to capture weekly intake patterns of foods that align with evidence-based dietary recommendations for chronic disease prevention. Seven of the twelve items correspond closely in construct and behavioural target to items included in the *Starting the Conversation* (STC) brief dietary assessment tool, a validated measure designed for use in primary care to assess diet quality and facilitate brief lifestyle counselling (Paxton et al., 2011).[31] Specifically, both instruments assess:

1. Intake of vegetables
2. Intake of fruits
3. Consumption of nuts and healthier fats
4. Consumption of sugary beverages
5. Consumption of fast foods
6. Intake of sweets and desserts
7. Consumption of snack chips/crackers

These overlapping items ensure that the PC-PEP dietary score reflects domains that are well established in short-form dietary assessment and associated with diet quality and chronic disease risk.

In addition to these seven items, five supplementary questions were included to align the measure with Canada's Food Guide,[30] which emphasizes:

- Increased intake of plant-based proteins (beans, lentils, fish, poultry)
- Preference for healthy unsaturated fats (e.g., extra virgin olive oil)
- Limitation of red meat consumption
- Reduction of processed meats
- Minimizing saturated fats (butter, margarine, meat fat)

These additions broaden the measure's relevance for prostate cancer survivorship and cardiometabolic prevention by capturing dietary behaviours directly targeted in national nutrition guidance.

Scoring Procedures

To ensure all items contributed in the same direction, items representing healthier choices (e.g., fruits, vegetables, nuts) were reverse-coded so that higher values reflect poorer dietary quality. Remaining items were forward coded after standardizing minimum values to zero. Depending on response options, individual item scores ranged from 0–3 or 0–5 and were summed to create a total diet summary score, with higher scores indicating less favourable dietary behaviour.

A complete list of questionnaire items and scoring rules appears below.

Dietary Questions

Table S1. Dietary questions used in summary score

Question	Responses options
Over the past 7 days, how many servings of vegetables (fresh or cooked, not fried) did you eat EACH DAY on average? 1 serving is the equivalent of 1 cup of vegetables.	1, None<br>2, less than 2 servings<br>3, 2-4 servings<br>4, 5 or more servings each day, on average over the past week
Over the past 7 days, how many servings of fruit (1 cup) did you eat EACH DAY on average?	1, None<br>2, less than 2 servings<br>3, 2-4 servings<br>4, 5 or more servings each day, on average over the past week
Over the past 7 days, how many servings (1 serving is the equivalent of 30 grams of mixture of peanuts, Brazilian nuts, walnuts, almonds, or hazelnuts) of nuts did you eat EACH DAY on average?	1, I did not eat any nuts this week<br>2, 2 or less times<br>3, 3-4 times<br>4, 5 or more times
Over the past 7 days, how many spoons of extra virgin olive oil did you consume EACH DAY on average?	1, None<br>2, 3 or less<br>3, 4 or more times on average, each day
Over the past 7 days, how many times did you eat beans (like lentils, peas, pinto, or black beans), chicken, white - not red - meat, fish EACH DAY on average?	1, None<br>2, less than 1 time<br>3, 1-2 times<br>4, 3 to 5 times<br>5, more than 5 times on average, each day
Over the past 7 days, how many times did you eat red meat?	1, I did not eat any red meat this week<br>2, 1-2 times this week<br>3, 3-4 times this week<br>4, 5 or more times this week
Over the past 7 days, how many times did you eat fast food meals or snacks EACH DAY on average?	1, I did not consume any fast food means or snacks<br>2, less than 2 times a day<br>3, 2 times<br>4, 3-4 times<br>5, 5 more times
This week, how many regular sodas and glasses of sweet tea did you drink EACH DAY on average?	1, None<br>2, 1 time or less<br>3, 2-3 times<br>4, 4 or more times

This week, how many times did you eat regular snack chips or crackers (not low fat) EACH DAY on average?	1, None<br>2, 1 time or less<br>3, 2-3 times<br>4, 4 or more times
This week, how many times did you eat commercial bakery goods, desserts, sweets, or pastries (not the low-fat kind) EACH DAY on average?	1, None<br>2, 1 time or less<br>3, 2-3 times<br>4, 4 or more times
This week, how much margarine, butter, or meat fat did you use to season vegetables or put on potatoes EACH DAY on average?	1, None<br>2, very little (half-a-spoon or less)<br>3, some (between half-spoon and a spoon-and-a-half)<br>4, a lot (more than a spoon-and-a-half)
Over the past 7 days, how many times did you eat processed meats EACH DAY on average?	1, None<br>2, 100 grams or less<br>3, More than 100 grams

Notes: To ensure all items reflected healthier dietary patterns in the same direction, the five items representing foods associated with healthier diet quality were reverse-coded (i.e., higher scores indicating unhealthy choices), while the remaining items were forward-coded by standardizing their minimum values to zero. Resulting item scores ranged between 0-3 to 0-5 depending on the response options.

Parallel Mediation Analysis

Table S2. Parallel mediation of the effect of PC-PEP vs waitlist control on weight at 6 months

	**Total**			**Direct**			**Indirect**		**% Mediated**
**Mediator**	**Effect Size (95% CI)**	**SE**	**p**	**Effect Size (95% CI)**	**SE**	**p**	**Effect Size (95% CI)**	**SE**	
Diet Summary Score	-3.65 (-5.63, -1.67)	1.00	<0.001	-2.44 (-4.55, -0.33)	1.07	0.024	-0.70 (-1.84, 0.03)	0.47	.
Physical Activity Level							-0.52 (-1.45, 0.08)	0.32	14%
Total Mediation							-1.21 (-2.49, -0.30)	0.55	33%

Notes: SE = standard error. CI = confidence interval. Mediation analysis was adjusted for prognostic covariates (age, Charleson Comorbidity Index, time between randomization and treatment, relationship status, medication for depression or anxiety), and baseline weight, diet, and physical activity level. n = 128.

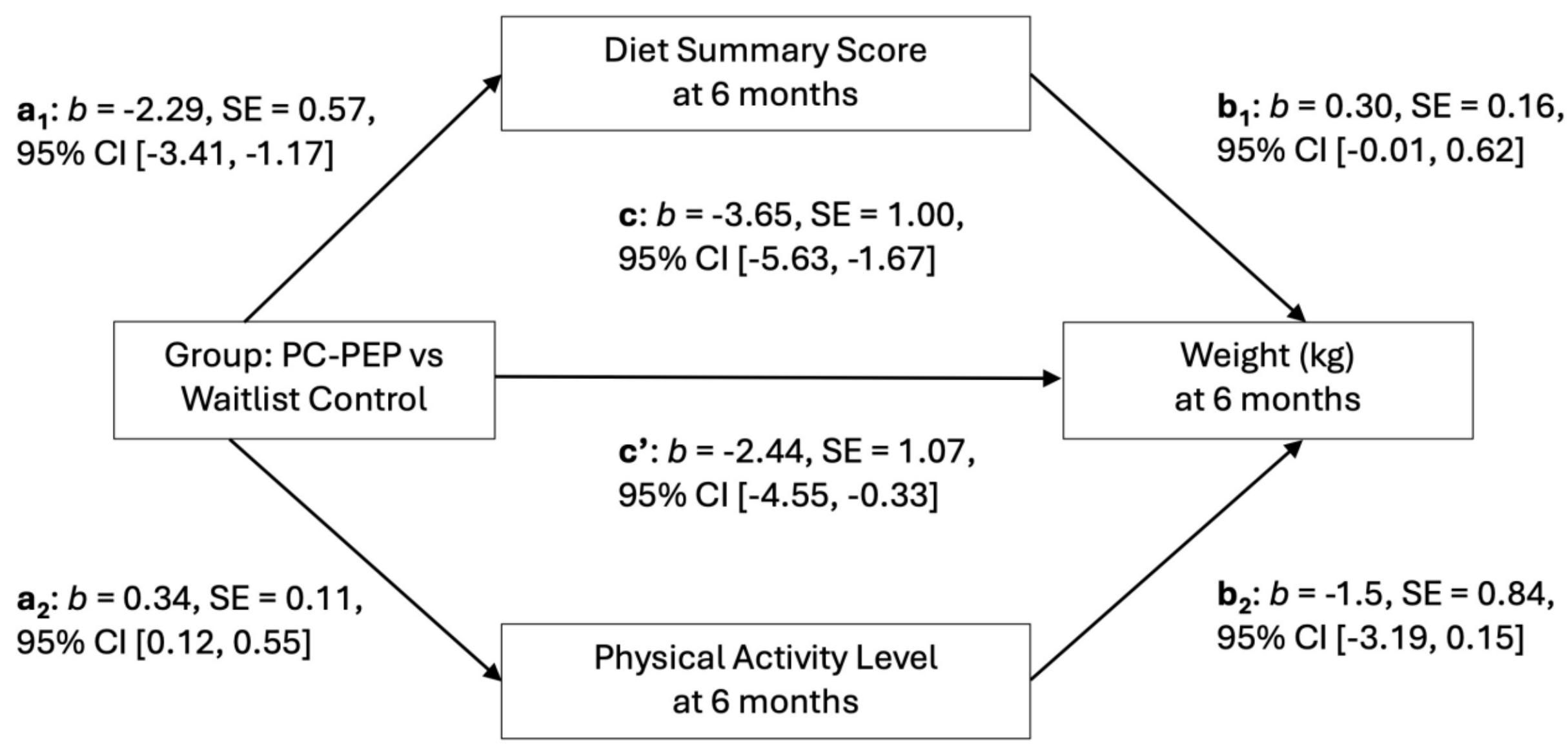

Figure S1. Parallel mediation of the Prostate Cancer Patient Empowerment Program (PC-PEP) on weight by diet summary score and physical activity level at 6 months.

Notes: b = regression coefficient. SE = standard error. CI = confidence interval. Mediation analyses were adjusted for age, Charleson Comorbidity Index, treatment modality, time between randomization and treatment, relationship status, medication for depression or anxiety, baseline weight, diet, and physical activity. n = 128.

Early vs Late Intervention Mediation Analysis

Table S3. Pre- and post-Intervention weight, diet scores, and physical activity, and during-program aerobic exercise and strength training

	PC-PEP (Early) n = 66	Waitlist Control (Late) n = 62	p-value
Pre-Intervention Mediator and Outcome Scores			
Weight (kg)	66, 85 (78, 96)	62, 84 (78, 96)	0.6
Diet Summary Score	66, 13 (11, 16)	62, 14 (10, 16)	0.7
Physical Activity			
Not Very Active (<30 min. per week)	11 (17%)	14 (23%)	0.7
Moderately Active (30-150 min. per week)	33 (50%)	27 (44%)	
Very Active (150+ min. per week)	22 (33%)	21 (34%)	
Post-Intervention Mediator and Outcomes Scores			
Weight (kg)	66, 84 (76, 94)	62, 82 (77, 93)	1.0
Diet Summary Score	66, 11 (9, 14)	62, 12 (10, 15)	0.2
Physical Activity			0.9
Not Very Active (<30 min. per week)	6 (9%)	7 (11%)	
Moderately Active (30-150 min. per week)	26 (39%)	25 (40%)	
Very Active (150+ min. per week)	34 (52%)	30 (48%)	
During-Intervention Exercise Duration			
Avg. Weekly Aerobic Exercise (min.)	66, 183 (109, 272)	50, 169 (99, 248)	0.5
Avg. Weekly Strength Training (min.)	66, 62 (43, 88)	50, 61 (32, 115)	0.6

Notes. Summary statistics are reported as n, median (25% to 75% range) for continuous variables, and n (%) for categorical variables. Pre-intervention is baseline for the early PC-PEP group and 6 months for the late group. Post-intervention is 6 months for the early group, and 12 months for the late group.

Table S4. Mediation of the effect of early vs late intervention on weight

	Total			Direct			Indirect	
**Mediator**	**Effect Size (95% CI)**	**SE**	**p**	**Effect Size (95% CI)**	**SE**	**p**	**Effect Size (95% CI)**	**SE**
Diet Summary Score	-0.65 (-3.84, 2.54)	1.61	0.70	-0.04 (-3.00, 2.93)	1.50	0.98	-0.61 (-2.12, 0.49)	0.64
Physical Activity Level	-0.63 (-3.84, 2.57)	1.62	0.70	-0.45 (-3.58, 2.69)	1.58	0.78	-0.19 (-1.07, 0.54)	0.39
Avg. weekly aerobic exercise (min)	-0.13 (-3.55 3.29)	1.72	0.94	-0.07 (-3.51, 3.37)	1.74	0.97	-0.05 (-0.93, 0.31)	0.27
Avg. weekly strength training (min)	-0.13 (-3.55, 3.29)	1.72	0.94	-0.14 (-3.56, 3.28)	1.73	0.94	0.01 (-0.35, 0.57)	0.21

Notes: SE = standard error. CI = confidence interval. Mediation analysis was adjusted for prognostic covariates (age, Charleson Comorbidity Index, time between randomization and treatment, relationship status, medication for depression or anxiety), pre-intervention weight, and pre-intervention diet or physical activity measure (to match the respective model). n = 128 for diet summary and physical activity level, n = 116 for avg. weekly aerobic exercise and strength training.
